# Biosynthesis and Biotechnological Synthesis of Hydroxytyrosol

**DOI:** 10.3390/foods13111694

**Published:** 2024-05-28

**Authors:** Jiali Tang, Jiaying Wang, Pengfei Gong, Haijing Zhang, Mengyao Zhang, Chenchen Qi, Guohui Chen, Chengtao Wang, Wei Chen

**Affiliations:** 1 Key Laboratory of Geriatric Nutrition and Health, Ministry of Education, Beijing Advanced Innovation Center for Food Nutrition and Human Health, Beijing Engineering and Technology Research Center of Food Additives, School of Food and Health, Beijing Technology and Business University, Beijing 100048, China; 2230202138@st.btbu.edu.cn (J.T.); 2230202145@st.btbu.edu.cn (J.W.); 2130021003@st.btbu.edu.cn (P.G.); haijingvv@yeah.net (H.Z.); 2230201051@st.btbu.edu.cn (M.Z.); wangchengtao@th.btbu.edu.cn (C.W.); 2 ACK Co., Ltd., Urumqi 830022, China; qichenchenxj@163.com (C.Q.); chenguohui@tsinkaz.com (G.C.)

**Keywords:** olive leaves, synthetic biology, nutraceutical, sustainable production, industrial applications

## Abstract

Hydroxytyrosol (HT), a plant-derived phenolic compound, is recognized for its potent antioxidant capabilities alongside a spectrum of pharmacological benefits, including anti-inflammatory, anti-cancer, anti-bacterial, and anti-viral properties. These attributes have propelled HT into the spotlight as a premier nutraceutical and food additive, heralding a new era in health and wellness applications. Traditional methods for HT production, encompassing physico-chemical techniques and plant extraction, are increasingly being supplanted by biotechnological approaches. These modern methodologies offer several advantages, notably environmental sustainability, safety, and cost-effectiveness, which align with current demands for green and efficient production processes. This review delves into the biosynthetic pathways of HT, highlighting the enzymatic steps involved and the pivotal role of genetic and metabolic engineering in enhancing HT yield. It also surveys the latest progress in the biotechnological synthesis of HT, examining innovative strategies that leverage both genetically modified and non-modified organisms. Furthermore, this review explores the burgeoning potential of HT as a nutraceutical, underscoring its diverse applications and the implications for human health. Through a detailed examination of both the biosynthesis and biotechnological advances in HT production, this review contributes valuable insights to the field, charting a course towards the sustainable and scalable production of this multifaceted compound.

## 1. Introduction

**Figure 1:**
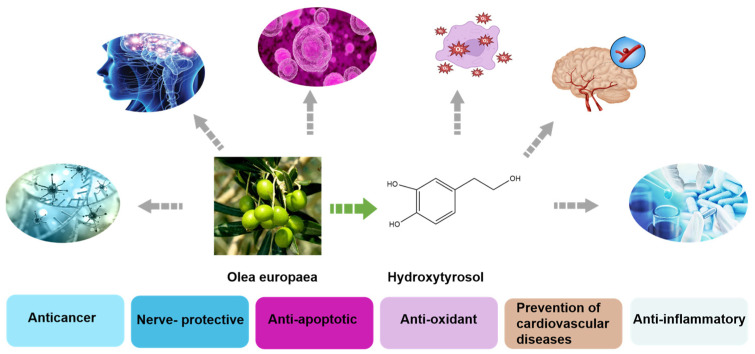
Functions of HT.

Hydroxytyrosol (HT), recognized as the most potent and efficient antioxidant known, is abundant in olives [1]. In nature, HT is present in the olive plant as part of the secoiridoid compound oleuropein found in its leaves, fruit, oil, and oil production waste products. The “Mediterranean Diet” [2], named after the countries around the Mediterranean Sea (Spain, Italy, France, and Greece), is a healthy, light, simple, and nutritious style of eating. As olive oil [3] is a prime constituent of the health-promoting Mediterranean diet, HT has obtained recognition for its attributes, supported by a recent health claim of the European Food Safety Authority. Currently, HT is used in cosmetics and food supplements, where it has anti-cancer, anti-inflammatory, anti-apoptotic, and neuroprotective activities and has been approved as a food additive by FDA GRAS. As a low-calorie alternative to sugar additives, HT [4] is often added to beverages, candies, chocolates, pastries, and other foods (Figure 1).

At present, HT can be synthesized through physico-chemical and biotechnological methods. Plant extraction [4,5,6] involves the isolation of oleuropein from olive oil production waste or olive branches and leaves, followed by acid or catalytic hydrolysis to extract HT. Chemical synthesis [1,7,8,9] is achieved under normal temperature conditions using solvents such as acetonitrile, tetrahydrofuran, dichloroethane, methylene chloride, or toluene. These solvents, along with 3,4-dimethoxyphenylethanol and aluminum triiodide, are mixed, followed by the addition of boron trifluoride tetrahydrofuran or boron trifluoride ether. Subsequently, the mixture is subjected to warming reflux, cooling, filtration, and activated carbon treatment to obtain white solid powdered HT. Biotechnological methods encompass enzymatic transformation, non-genetically engineered bacteria, and genetically engineered bacteria for HT production.

Fernandez-Bolanos et al. [10] investigated the hydrothermal treatment of two-phase olive waste (alperujo) and its effect on HT solubility. They successfully extracted and purified HT from alperujo using an inexpensive chromatographic system, yielding approximately 4.5–5 kg of HT from 1000 kg of alperujo with a moisture content of 70%. Following purification, a minimum of 3 kg of HT with a purity of 90–95% was obtained. Neji et al. [11] catalyzed the generation of HT extracts from tyrosol through a wet hydrogen peroxide-catalyzed oxidation reaction using montmorillonite K-Catalyst (KSF) as a solid acid catalyst at room temperature. Papageorgiou et al. [12] employed a solid–liquid extraction method to obtain HT from oleuropein, utilizing a water–ethanol mixture, hydrochloric acid hydrolysis, and optimized ethyl acetate extraction. They recovered 10–15 g of HT per kg of olive leaves. Ben Amor et al. [13] used an ethanol–water mixture (50:50) as the optimal solvent for the extraction of bioactive substances from olive pomace, from olive oil pomace enriched with 5% additives, to extract bioactive molecules with HT as the main component (43%). Squillaci et al. [14] obtained acidic extracts rich in HT from olive oil dregs by incubating them at 37 degrees C for 1 h under pH 1.25 conditions. The recovery of HT was achieved to 92.50% using Amberlite XAD16N and XAD7HP resins with 25% ethanol as the optimal elution condition.

While plant extraction and chemical synthesis [1,4] methods have certain drawbacks, such as low recovery, lengthy cycle times, tedious steps, high costs, and environmental pollution due to the use of organic reagents, biotechnological synthesis of HT demonstrates advantages in terms of being environmentally friendly, safe, and cost-effective. The development of biological methods is rapidly gaining popularity and is expected to become mainstream in the future. The biotechnological synthesis of HT, in its ascendancy, embodies the confluence of innovation and tradition, harnessing the genetic and metabolic capabilities of microorganisms to forge a sustainable path for HT production. This methodological evolution [15,16,17,18] from plant extraction and chemical synthesis towards biotechnological processes underscores a broader shift towards environmental stewardship and efficiency. The advancement of biological methods, poised at the frontier of HT synthesis, not only promises to refine the production process but also to broaden the horizons for HT application, embedding it more deeply into the fabric of nutritional, pharmaceutical, and cosmetic industries. As we delve into the intricacies of HT’s biosynthesis and biotechnological production, the journey from the olive grove to the laboratory encapsulates a broader narrative of scientific endeavor and innovation. The exploration of HT’s biosynthetic pathways, the engineering of microbial factories, and the optimization of production processes collectively illuminate the potential of biotechnology to transcend traditional boundaries and catalyze a new era of sustainable production. This transition, underpinned by rigorous research and a commitment to sustainability, not only heralds a new chapter in the story of HT but also reflects a broader paradigm shift towards greener and more efficient production methodologies in the biotechnological arena.

In conclusion, this chapter explores the synthesis [10,11,12] and application of HT, tracing its progression from historical methods in ancient olive cultivation to modern biotechnological advancements. The development of HT production techniques reflects a significant shift towards sustainable and efficient scientific practices. This evolution underscores the integration of traditional approaches with cutting-edge science, highlighting their collective impact on the field of nutraceuticals. As we further our understanding of HT’s biosynthesis and continue to refine biotechnological synthesis methods, we are poised to expand its applications, reinforcing its vital role in promoting health and well-being in the contemporary era. This chapter sets the stage for future research that could unlock additional therapeutic potentials of HT, emphasizing its importance in the ongoing pursuit of sustainable health solutions.

## 2. Natural Biosynthetic Pathway of HT

The biosynthesis of HT involves a complex network of enzymes that have not yet been fully elucidated. Guodong et al. [19] employed a combined Societe Generale de Surveillance (SGS) and Single Molecule Real-Time (SMRT) sequencing approach to analyze the genes associated with the HT biosynthesis pathway, revealing that several enzymes, including polyphenol oxidase (PPO), DOPA decarboxylase (DDC), copper amine oxidase (CuAO), and acetaldehyde dehydrogenase (ALDH), play crucial roles. Mougiou et al. [20] conducted transcriptome analysis of young olive fruits, demonstrating the presence of transcripts for all enzymes involved in the HT biosynthesis pathway. Enzymatic activities of tyrosine decarboxylase TDC, CuAO, ALDH, and PPO were identified from the proposed HT biosynthesis genes (*tdc*, *mao*, *par*, *tyr*, *th*, *ddc*, *aldh*). These findings revealed that two HT biosynthetic pathways can be formed using tyrosine as a precursor molecule. In one pathway, tyrosine is converted to L-DOPA by PPO and then to dopamine by TDC; subsequently, CuAO generates 3,4-dihydroxyphenylacetaldehyde (3,4-DHPA), which ultimately leads to HT formation through ALDH. In the other pathway, tyrosine is transformed into tyramine by TDC, followed by CuAO-mediated conversion to 4-hydroxyphenylacetic acid (4-HPA). Then, ALDH produces tyrosol, which is further converted to HT by PPO. Sanchez et al. [21] identified two phenylethylaldehyde reductase genes, OePAR1.1 and OePAR1.2, involved in HT synthesis. The reaction catalyzed by OePAR is an important biochemical step in the formation of HT from the amino acid L-3,4-dihydroxyphenylalanine (L-DOPA) in olives.

Beyond the olive tree, the quest to understand HT biosynthesis [22,23,24] extends into the realm of microbial fermentation. During alcoholic fermentation, various heteroalcohols are believed to be generated through amino acid metabolism pathways. These pathways include the deamination of amino acids to α-keto acids, followed by decarboxylation to aldehydes and subsequent reduction to heteroalcohols (Ehrlich pathway). Additionally, α-keto acids produced during amino acid biosynthesis from sugars can undergo degradation to form heteroalcohols. Gallardo-Fernandez et al. [25] employed isotopic labeling analysis to investigate HT biosynthesis in *Saccharomyces cerevisiae*. The study revealed the presence of unlabeled compounds alongside tyrosine, suggesting the existence of an alternative pathway for HT synthesis. Moreover, the concentration of unlabeled HT was approximately ten times higher than that of labeled HT, indicating that both the mangiferylic acid pathway and the Ehrlich pathway contribute to HT formation, with the former playing a more prominent role in tyrosol synthesis. Consequently, sugar metabolism emerges as the primary precursor for the synthesis of aromatic higher alcohols. This research not only expands our understanding of HT synthesis in different biological systems but also opens up possibilities for biotechnological production of HT, leveraging microbial fermentation processes.

The influence of environmental factors on HT biosynthesis has also been a focal point of recent studies. In the context of low-temperature stress on olive trees and its relationship with genotype, Mougiou et al. [26] investigated the physiological and biochemical effects. Their findings demonstrated that mRNA levels of PPO genes involved in HT biosynthesis and plant defense were upregulated after 24 h of stress at 0 °C, with elevated expression persisting for an extended duration. Notably, three genes involved in HT biosynthesis exhibited increased expression levels following cold stress, suggesting that low-temperature conditions induce prolonged upregulation of mRNA and related genes associated with HT biosynthesis. This insight not only enriches our understanding of HT biosynthesis but also offers practical implications for agricultural practices aimed at enhancing HT content in olive crops.

In conclusion, the biosynthetic pathways of HT exemplify the sophistication inherent in natural product synthesis. The orchestration of various enzymes, coupled with the identification of critical genes and the modulation by environmental factors, underscores the complexity of HT production. A thorough understanding of these pathways is crucial, not only for optimizing the biotechnological production of HT but also for enhancing the health-promoting qualities of olive-based products. Ongoing research into HT is progressively revealing ways to harness scientific advancements for novel production techniques. This ongoing exploration is poised to position HT as a cornerstone in the future of nutraceutical innovation, emphasizing its potential in enhancing human health through dietary sources.

## 3. Biotransformation via Enzymes

Enzymatic biotransformation stands at the forefront of innovative methods for HT production, leveraging the specificity and efficiency of enzymes to convert structurally similar substrates into valuable compounds. This approach has garnered attention for its potential to offer a green, sustainable alternative to chemical synthesis, reducing the environmental footprint and enhancing the purity of the final product. The investigation of diverse enzymes, including fungal glucosidases and marine *α*-glucosidases, reveals multiple viable pathways for HT synthesis. Each pathway presents distinct advantages and challenges, highlighting the potential for tailored approaches in biotechnological applications. Several investigations have explored the utilization of diverse enzymes and substrates to achieve efficient HT synthesis. Hamza and Sayadi [27] employed *Aspergillus niger-glucosidase* generated by A. *niger* to catalyze the bioconversion of rape leaf extract and olive mill wastewater, resulting in the extraction of HT, with maximum concentrations of 1.1 and 0.5 g/L. They meticulously optimized the submerged culture conditions and devised parameters for the effective production of the enzyme. This not only addresses waste disposal issues but also adds value to by-products of the olive oil industry. The meticulous optimization of culture conditions underlines the importance of tailored environmental parameters in maximizing enzyme productivity and efficiency. Chatzikonstantinou et al. [28] developed a potent biocatalyst by immobilizing β-glucosidase on chitosan-coated magnetic beads. This biocatalyst facilitated the modification of olive leaf extracts, attaining notable conversion of oleuropein (exceeding 90%) and a 2.5-fold enrichment of HT. Macedo et al. [29] integrated microwave treatment and enzymatic extraction to extract phenolic and antioxidant-rich extracts from olive pomace. The results showed that the extraction conditions combining microwave treatment with pectinase, cellulase, and tannase were more favorable for the release of HT. The yield of this HT can reach 59.29 (mg/kg of pomace).

Briante et al. [30] immobilized partially purified hyperthermophilic β-glycosidase on chitosan support, thereby enabling the expedited biotransformation of oleaginous leaf extract into highly purified HT (91–94% in weight) within a short duration (14–16 h). The use of such enzymes can significantly reduce the risk of microbial contamination, a common concern in biotechnological processes, and can increase reaction rates, leading to more efficient production processes. Trincone et al. [31] synthetized novel HT mono- and disaccharide derivatives through the enzymatic conversion of tyrosol glycoside derivatives utilizing marine α-glucosidase from *Aplysia fasciata* and commercial tyrosinase from mushrooms. This methodology yielded HT products with final concentrations of 9.35 and 10.8 g/L, respectively.

Furthermore, engineered enzymes have been employed to enhance HT synthesis. Brouk et al. [32] harnessed protein engineering and statistical modeling to enhance the substrate specificity and oxidative activity of toluene monooxygenase. This approach enabled the efficient synthesis of HT from abundant and cost-effective 2-phenylethanol. This not only makes the process more economically viable but also opens up new pathways for the synthesis of HT and related compounds. Donadio et al. [33] investigated the biotransformation of unconventional substrates, including 2-phenoxyethanol, phthalate, and 2-indanol, utilizing recombinant toluene o-xylene monooxygenase expressed in *Escherichia coli* cells. Notably, they successfully generated six hydroxylated derivatives, including HT, from these substrates. This approach not only broadens the scope of substrates that can be utilized but also demonstrates the potential of genetic engineering in creating tailor-made enzymes for specific biotransformation tasks.

To surmount the limitations associated with the conversion of tyrosol to HT by tyrosinase, Deri-Zenaty et al. [9] developed a continuous dual enzyme reaction system utilizing sol–gel-immobilized tyrosinase from *Bacillus megaterium* and glucose dehydrogenase expressed in *E. coli* cell extract. Under optimized conditions, this system yielded HT with a final concentration of 7.68 g/L and a productivity of 2.30 mg of HT/mg of TyrBm bead. By optimizing the conditions for the conversion of tyrosol to HT, this system highlights the potential of integrating multiple enzymatic steps into a single, continuous process, thereby enhancing productivity and scalability.

The collective efforts of researchers in the field of enzymatic biotransformation have laid a solid foundation for the sustainable production of HT. Each study contributes to a growing body of knowledge that not only advances our understanding of enzyme-catalyzed reactions but also brings us closer to realizing the full industrial potential of enzymatic HT production. Looking forward, the further refinement of enzyme systems, through protein engineering and process optimization, holds great promise for enhancing HT yields and productivity. Additionally, the exploration of novel enzymatic pathways and the development of more robust and versatile biocatalysts will undoubtedly open new avenues for the efficient and eco-friendly production of HT.

In conclusion, enzymatic biotransformation stands out as a pivotal approach in advancing sustainable production of HT. This method exemplifies the integration of green chemistry principles with industrial biotechnology, emphasizing the role of enzymes in achieving specificity, efficiency, and adaptability in HT synthesis. Through continued advancements in enzyme engineering and bioprocess optimization, we are paving a path towards a future where HT and other phenolic compounds are produced in a manner that is both environmentally responsible and economically viable. The ongoing exploration and enhancement of enzymatic capabilities are opening new avenues for innovative, sustainable production strategies in the nutraceutical industry, heralding a more sustainable and prosperous future.

## 4. Production via Non-Genetic Modification Organisms (Non-GMOs)

The exploration of non-GMOs for the production of HT represents a pivotal shift towards more sustainable and publicly acceptable methods in biotechnological synthesis. This innovative approach leverages the intrinsic metabolic pathways of various microbial strains, offering a greener alternative to genetically modified counterparts. The utilization of such organisms, coupled with advanced immobilization techniques, underscores a commitment to environmental sustainability and safety, aligning with the growing consumer demand for naturally derived compounds.

**Figure 2:**
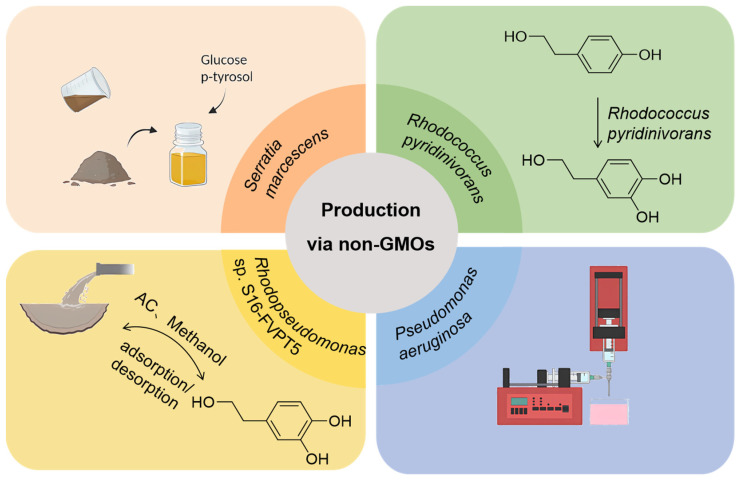
Production of HT by non-genetically engineered bacteria (adapted from Bouallagui and Sayadi [35], Carlozzi et al. [36], Rebollo-Romero et al. [37], and Anissi et al. [38]).

Researchers have explored non-GMO approaches for the production of HT using various microbial strains and immobilization techniques (Figure 2). Allouche and Sayadi [34] conducted a screening of the *Serratia marcescens* strain using *p*-tyrosol as the sole carbon source. They optimized the growth conditions and *p*-tyrosol concentration for the conversion of *p*-tyrosol to HT during bacterial growth. The best HT yield (80%) was obtained by *S. marcescens* growing cells at the end of the exponential phase with the addition of 2 g/L of *p*-tyrosol followed by 1 g/L of *p*-tyrosol after 7 h of incubation. This study not only highlights the efficiency of *S. marcescens* in converting *p*-tyrosol to HT but also sets a precedent for the potential exploitation of similar bacterial strains in biotechnological applications. Bouallagui and Sayadi [35] employed immobilized resting cells of *Pseudomonas aeruginosa* in calcium alginate beads to enhance HT production. The biotransformation rate of cells immobilized in calcium alginate beads reached 86% in the presence of 5 g/L tyrosol during a single batch process. This method represents a significant advancement in biocatalyst design, offering a scalable and efficient approach to HT production that could be adapted to various microbial systems. Carlozzi et al. [36] produced a sustainable and environmentally friendly product from olive oil mill effluent by using residual effluent as a feedstock and utilizing *Rhodopseudomonas* sp. S16-FVPT5 to obtain an HT-rich mixture. This approach not only addresses environmental concerns associated with olive mill waste but also contributes to the circular economy by converting waste into valuable products. The success of this strategy highlights the versatility of non-GMOs in bioremediation and biotransformation processes, opening new avenues for the utilization of agro-industrial waste. Rebollo-Romero et al. [37] investigated factors influencing HT yield during alcoholic fermentation, including yeast strain, initial tyrosine concentration as a precursor, and the effect of synthetic and sterilized natural grape juice. Commercially available yeast resulted in a higher final HT yield, reaching a maximum of 6.12 ng/mL. This study sheds light on the complex interplay between microbial metabolism and product formation. The findings suggest that the selection of suitable yeast strains and optimization of fermentation conditions could significantly enhance HT production, offering a promising avenue for large-scale synthesis. Anissi et al. [38] focused on a Gram-positive bacterium capable of producing HT through the conversion of tyrosol or L-tyrosine. They identified the bacterium as *R. pyridinivorans* strain 3HYL DSM109178 based on phenotypic characteristics and 16S rDNA sequence. They also developed a plasmid-cured strain, *R. pyridinivorans* 3HYL-AO, through random chemical mutagenesis. The wild-type strain yielded 16.4 +/− 0.23 mmol/L of HT from tyrosol, while the mutant strain *R. pyridinivorans* 3HYL-AO produced 21.75 +/− 0.34 mmol/L of HT. This study not only demonstrates the potential of *R. pyridinivorans* in HT production but also illustrates the feasibility of non-genetic approaches to microbial strain enhancement.

Collectively, these studies highlight the efficacy of non-GMO techniques in the biotechnological production of HT. Utilizing the inherent metabolic capacities of diverse microbial strains, alongside state-of-the-art bioprocessing technologies, researchers have successfully demonstrated the sustainable, efficient, and scalable production of HT. The promising outcomes of these methodologies underscore the necessity for continued investigation and enhancement of non-GMO strategies in HT synthesis. Future research could focus on identifying additional microbial strains with innate or improved abilities to produce HT, developing more effective enzyme immobilization techniques, and refining fermentation protocols. With ongoing advancements and meticulous optimization, non-GMO methods are poised to significantly contribute to the sustainable and eco-friendly manufacturing of HT and other essential nutraceutical compounds.

## 5. Production via GMOs

The exploration of GMOs for the biosynthesis of HT represents a significant leap forward in the field of biotechnology. *E. coli* possesses several advantageous [39,40] characteristics for the biosynthesis of HT, including a well-established genetic background, high expression levels of target genes, a robust expression system, ease of cultivation, rapid growth, strong resistance to contamination, and low cost. Furthermore, *E. coli* has been approved by the U.S. FDA as a safe genetically engineered host organism. As a result, it has found extensive use in HT biosynthesis (Table 1).

The biosynthesis of HT can be hindered due to the through conversion of HT to *ortho*-quinone by tyrosinase or the lack of cofactors necessary for tyrosine hydroxylase. To address these challenges, researchers have explored various strategies. For instance, Satoh et al. [41] demonstrated that the cofactor tetrahydroadenine (MH_4_) from *E. coli* could act as an alternative cofactor to TH in the presence of the BH4 regeneration pathway, preventing overoxidation. Additionally, the knockout of endogenous aromatic aldehyde oxidase was employed to prevent the formation of by-products, resulting in the nearly complete synthesis of HT in engineered *E. coli*. Other studies [42,43] have utilized *E. coli* strains carrying specific hydroxylases to synthesize HT, resulting in notable yields of HT through strain optimization.

**Table 1:** Synthesis of HT production using Genetic Modification Organisms described in the literature.

Modification Organisms	Substrate	Amount of Substrate	Experimental Process	Amount of Product	Reference
*E. coli*	L-Tyrosine	1 mM	An artificial pathway for L-tyrosine oxidation was introduced into *Escherichia coli* using mammalian tyrosine hydroxylase (TH) and an endogenous cofactor in Escherichia coli (MH_4_) and knocking out endogenous aromatic aldehyde oxidase.	0.19 mM	[41]
*E. coli*	Tyrosine	15 mM	Obtaining the hybrid hydroxylase HpaBC for production applications through protein engineering and directed divergent evolutionary strategies.	93%	[44]
*E. coli*	Tyrosine	3 mM	Replacement of mouse tyrosine hydroxylase by HpaBC from *Escherichia coli* using protein engineering and in vivo targeting optimization and design of VanR regulatory proteins as hydroxytyrosol biosensors.	95%	[45]
*E. coli*	L-Tyrosine	50 mM	An enzyme cascade consisting of HpaBC from *Escherichia coli*, L-amino acid deaminase (LAAD) from *Aspergillus singularis*, α-keto acid decarboxylase (ARO10) from *Saccharomyces cerevisiae*, and PAR from *S. lycopersicum* was designed.	97.1%	[46]
*B. licheniformis*	Glucose	80 g/L	Through protein engineering ketoacid decarboxylase, boosting the phosphoenolpyruvate (PEP) supply, releasing feedback inhibition, and blocking competing pathways.	9475 mg L/L	[47]
*S. cerevisiae*	Tyrosine/ Tyrosol	1 mM	HpaBC enzyme complex from *Escherichia coli* heterologously overexpressed in *Saccharomyces cerevisiae*.	1.15 mg/L/4.6 mg/L	[48]
*S. cerevisiae*	Glucose	160 g/L	Integration of the heterologous hydroxylase complex HpaBC from *Escherichia coli* into the genome of *Saccharomyces cerevisiae* shifts metabolism toward tyrosol synthesis.	375 mg/L	[49]
*S. cerevisiae*	Glucose		Overexpression of phenol hydroxylase; multimodal engineering approaches such as integrating the genomes of aro4(K229L) and aro7(G)(141S) to eliminate tyrosine feedback inhibition, constructing an AAS enzyme tyrosine metabolism pathway and incorporating an exogenous gene, Bbxfpk(op)(t), to distribute the flux, to enhance the supply of hydroxytyrosol precursors, and utilizing the GAL system to dynamically regulate hydroxytyrosol biosynthesis through carbon source regulation.	167.98 mg/g	[50]
*S. cerevisiae–E. coli*	Sucrose	10 g/L	De novo production of tyrosol using the *Saccharomyces cerevisiae* endogenous Ehrlich pathway, which converts tyrosol to hydroxytyrosol via Escherichia coli hydroxyphenylacetate 3-monooxygenase (EcHpaBC).	435.32 mg/L	[51]

In the pursuit of efficient HT production, Chen et al. [44] designed two biosynthetic pathways using engineered *E. coli* strains as biocatalysts, representing a pioneering effort in the field. Each pathway contained a catalytic step that lacked an efficient enzyme. Through protein engineering, highly active tyrosol hydroxylases or tyramine hydroxylases were obtained to complete the pathways, resulting in elevated HT yields compared to previously reported routes. Furthermore, a monooxygenase mutant with hybrid function was developed through directed divergent evolution, which led to a significant increase in HT yield. Notably, the complex pathway achieved a much higher HT yield (93%) compared to pathway 1 (56%) and pathway 2 (54%). By filling enzymatic gaps through protein engineering, they achieved unprecedented HT yields, showcasing the transformative power of genetic engineering in bioproduction processes. Furthermore, the creation of a monooxygenase mutant through directed evolution not only enhanced HT yield but also demonstrated the versatility and adaptability of engineered microbial systems.

**Figure 3:**
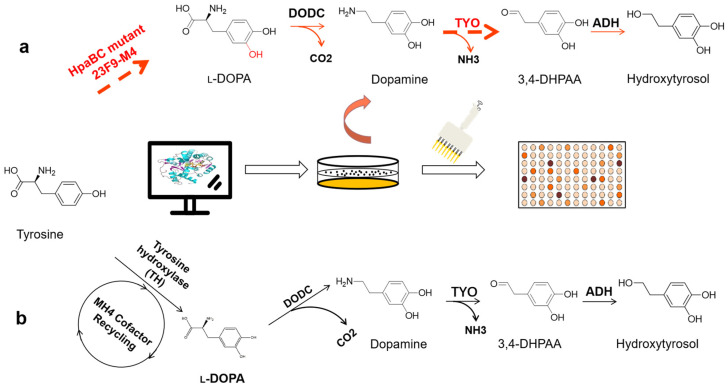
Pathway for oxidation of tyrosine to HT (adapted from Chen et al. [44]). (**a**) Highly efficient mutant enzymes HpaBC and TYO were obtained by protein engineering, which sequentially lifted the two rate-limiting steps in the original HT synthesis pathway and increased the HT synthesis capacity of the original synthesis pathway (conversion rate greater than 90%). (**b**) L-tyrosine oxidation is catalyzed by tyrosine hydroxylase (TH) in the presence of the pterin cofactor (conversion rate less than 20%). DODC: L-DOPA decarboxylase; TYO: tyramine oxidase; ADH: alcohol dehydrogenase; 3,4-DHPAA: 3,4-dihydroxyphenylacetaldehyde.

As shown in Figure 3, to overcome rate-limiting steps in HT biosynthesis, Yao et al. [45] developed efficient engineered *E. coli* by addressing the limiting enzymatic step. By replacing mouse tyrosine hydroxylase with the HpaBC monooxygenase from the *E. coli* BL21 (DE3) strain and optimizing tyramine oxidase activity using an HT-responsive biosensor, the conversion from tyrosine to HT was achieved at a remarkable efficiency of 95%. This strategic manipulation of enzymatic activity underscores the potential of synthetic biology to overcome natural limitations and significantly boost production capacities. Similarly, Zeng et al. [46] established an enzymatic cascade pathway for HT synthesis using L-tyrosine as a substrate. This pathway involved the coordinated action of HpaBC, L-amino acid deaminase (LAAD), α-keto acid decarboxylase (ARO10), and phenylalanine ammonia-lyase (PAL), resulting in high catalytic activity and efficient HT synthesis. Using this pathway, 24.27 mM of HT was obtained from 25 mM of L-tyrosine with a conversion rate of 97.1%.

Besides *E. coli*, other host organisms are gradually being used for HT production. Recent studies have applied Bacillus licheniformis to the production of HT, and *S. cerevisiae* has emerged as a safe and promising host organism for HT production. Zhan et al. [47] achieved overproduction of HT by engineering the HT biosynthesis pathway in Bacillus licheniformis, enhancing precursor supply, eliminating competing pathways, and modifying the glucose transport system, and they identified and eliminated several new rate-limiting steps by blocking tyrosol degradation, fine-tuning the expression levels of kivDV461I and hpaBC and overexpressing tkt and zwf. Finally, the best engineered strain, DHT23, produced 9475 mg L^−1^ of HT under fed-batch conditions. Muniz-Calvo et al. [48] improved HT production in *S. cerevisiae* by heterologously expressing the HpaBC enzyme complex from *E. coli*. Overexpression of hpaB and hpaC genes in *S. cerevisiae* led to enhanced HT production (3400 ± 500 μg/L), highlighting the potential of HpaBC overexpression as a tool to redirect central carbon flux towards tyrosine catabolism. Bisquert et al. [49] integrated the HpaBC hydroxylase complex into the genome of *S. cerevisiae*, resulting in increased HT productivity without the need for exogenous tyrosol or tyrosine supplementation (375 mg/L). The ability to enhance HT production without the need for exogenous substrates further illustrates the efficiency and sustainability of genetically engineered solutions. Liu et al. [50] also achieved substantial improvements in HT production by constructing new synthetic pathways in *S. cerevisiae* and optimizing key enzymatic steps (308.65 mg/L). These advancements resulted in the highest reported titer of HT biosynthesis in microorganisms to date (375 mg/L), showcasing the cutting-edge advancements being made in microbial engineering for nutraceutical production.

**Figure 4:**
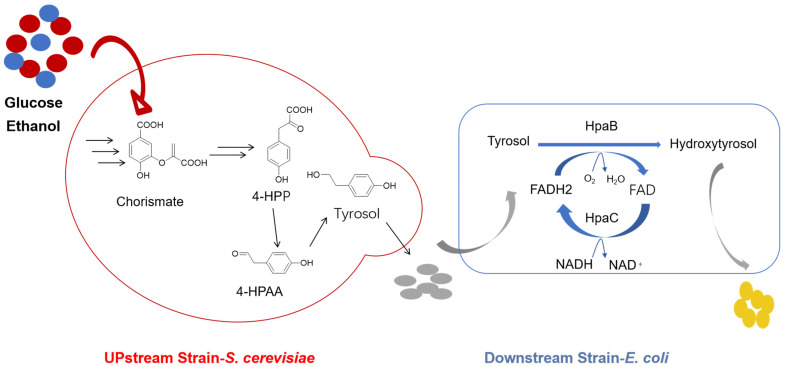
De novo biosynthesis of HT using a co-culture system consisting of *S. cerevisiae* and *E. coli* (adapted from Liu et al. [51]). 4-HPAA: 4-hydroxyphenylacetaldehyde; 4-HPP: 4-hydroxyphenylpyruvic; FAD: flavin adenine dinucleotide; FADH_2_: flavin adenine dinucleotide reduced; NADH: nicotinamide adenine dinucleotide.

Co-culture studies involving engineered strains have shown promise for the production of HT with enhanced efficiency and yield. One example of such research is the study conducted by Liu et al. [51], where a co-culture system consisting of *S. cerevisiae* and *E. coli* was developed to efficiently produce HT through de novo biosynthesis. The metabolic capabilities of both microorganisms were leveraged, with *S. cerevisiae* engineered to produce tyrosol using its endogenous Ehrlich pathway, and *E. coli* dedicated to converting tyrosol into HT using HpaBC. To optimize HT production, intra- and intermodule engineering strategies were implemented in this microbial consortium, leading to the production of 435.32 mg/L of HT (Figure 4). This approach not only highlights the synergistic potential of co-culturing strategies but also points to future directions in microbial consortium engineering for biomanufacturing. In another study, Gong et al. [43] developed a novel approach to improve the production of HT. The strategy involved designing a biosynthetic pathway using tyrosine as the substrate and selecting specific enzymes. Glutamate dehydrogenase GdhA was also overexpressed to facilitate cofactor cycling through coupled reactions catalyzed by transaminase and reductase. To optimize the process, the biosynthetic pathway was divided into two parts and utilized separate *E. coli* strains. This approach successfully achieved an HT yield of 9.2 mM from 10 mM tyrosine through careful optimization, illustrating the power of system-level engineering in biotechnology.

These studies demonstrate the potential of co-culturing engineered strains for efficient HT production. By strategically designing and combining the metabolic pathways of different strains, co-culture systems can effectively convert precursor compounds into HT, leading to higher yields. The utilization of engineered strains in co-culture systems holds promise for the development of robust and sustainable processes for HT production.

The advancements in GMO-based HT production underscore a broader trend in biomanufacturing, where the convergence of genetic engineering, synthetic biology, and systems biotechnology is opening unprecedented opportunities for the sustainable and efficient production of valuable compounds. The success of these approaches in enhancing HT yields not only demonstrates the feasibility of GMOs in industrial bioproduction but also sets the stage for future innovations in the field.

As research progresses, the integration of omics technologies, computational modeling, and machine learning will further refine our understanding of microbial metabolism and genetic regulation, enabling the design of even more efficient HT production systems. Additionally, the exploration of alternative microbial hosts, the development of more robust genetic tools, and the optimization of fermentation processes will continue to expand the capabilities and applications of genetically engineered microorganisms in nutraceutical production.

In conclusion, the utilization of GMOs for HT production signifies a transformative approach in biomanufacturing, providing scalable, sustainable, and economically feasible alternatives to conventional synthesis techniques. The breakthroughs in genetically engineering *E. coli* and *S. cerevisiae* underscore the substantial capabilities of genetic and metabolic engineering to maximize the commercial and therapeutic potential of HT. As the demand for HT and similar nutraceuticals continues to grow, further advancements and refinements in this field are essential. The sustained progress in GMO technologies is set to reinforce their pivotal role in the future landscape of biotechnological production, aligning with the needs of both industry and health care sectors.

## 6. Conclusions and Perspective

In conclusion, the biosynthesis and production of HT have made significant progress, offering valuable insights for industrial development. With a variety of properties and high value, HT is an attractive compound with potential for a wide range of applications.

**Figure 5:**
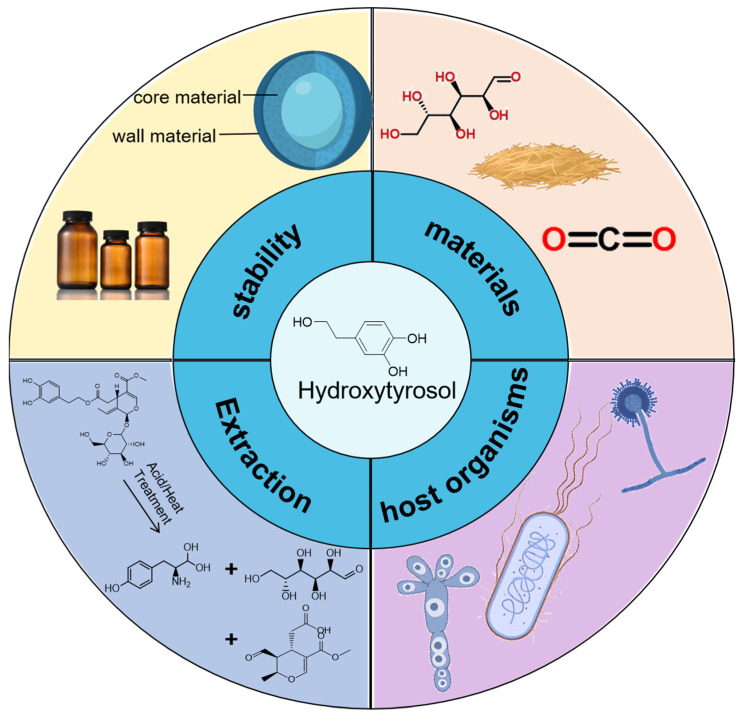
Applications and perspectives of HT.

Biological production methods, particularly through genetic modification of microorganisms, are rapidly advancing and offer advantages such as scalability and sustainability (Figure 5).

However, there are still challenges to address in the production of HT. The limited availability of suitable cell factories is a current limitation that needs to be overcome. Research efforts should focus on expanding the range of organisms that can efficiently produce HT, exploring alternative host organisms, and optimizing existing ones. Cost is another important consideration. Although biological production is advantageous in terms of scalability, the cost of substrates, such as glucose, can still be significant. Future efforts should explore the use of cheaper raw materials or waste streams as feedstocks for HT production, aiming to reduce production costs and enhance economic viability. Furthermore, the extraction and stability of HT are areas that require attention. Developing efficient extraction methods and improving the stability of HT during processing, storage, and utilization are crucial for its commercial application.

Overall, future research and development should focus on expanding the repertoire of cell factories, optimizing production processes, reducing costs, and addressing extraction and stability issues. With continued efforts and advancements in these areas, the industrial production of HT can be further improved, enabling its widespread utilization in various industries.

## Data Availability

No new data were created or analyzed in this study. Data sharing is not applicable to this article.

## Abbreviations

HT: hydroxytyrosol; KSFs: Key Success Factors; SGS: Societe Generale de Surveillance; SMRT: Single Molecule Real-Time; PPO: polyphenol oxidase; DDC: DOPA decarboxylase; CuAO: copper amine oxidase; ALDH: acetaldehyde dehydrogenase; 3,4-DHPA: 3,4-dihydroxyphenylacetaldehyde; 4-HPA: 4-hydroxyphenylacetic acid; L-DOPA: L-3,4-dihydroxyphenylalanine; GMOs: Genetic Modification Organisms.
